# Supplementary material for: Radiation-Induced Retinopathy and Optic Neuropathy after Radiation Therapy for Brain, Head, and Neck Tumors: A Systematic Review
Source: Cancers (Basel). 2023 Mar 27;15(7):1999. doi: 10.3390/cancers15071999 (PMC10093581; doi:10.3390/cancers15071999)
Supplement: Supplementary file 1 [file cancers-15-01999-s001.zip › cancers-2141852-supplementary.pdf]

Supplementary material

Table S1: Table showing which radiotherapy technique and dose per fraction was used in each study.

Study	Year	Radiation Technique	Mean Total Dose	Dose per fraction
Albano L	2019	MGKR	21	7-10
Amoaku WMK	1990	EBRT	47.3	2.4
Ares C	2009	PBRT	71.7	1.8-2.0
Astradsson A	2017	FSRT	54	1.8-2.0
Astradsson A	2014	FSRT	54	1.8-2.0
Bhandare N	2005	EBRT	NA	NA
Brecht S	2019	IMRT	60.5	NA
Claus F	2002	EBRT	NA	NA
Colin P	2005	FSRT	50.4	1.8
Demizu Y	2009	CIR/PBRT	57.6	3.6
Demizu Y	2009	PBRT	65	2.5
Duprez F	2012	IMRT	65	2.0
Ebara T	2019	EBRT	60	2.0
Elhateer H	2008	FSRT	50.4	1.8
Erridge S	2009	EBRT	45	1.8-2.5
Farzin M	2016	IMRT/3D/SRS	54	1.7-17
Flickinger JC	1989	EBRT	44.17	1.8
Flickinger JC	1990	EBRT	60	1.83
Fuji H	2011	EBRT	60	1.8
Hara W	2008	EBRT +SRT	66	2.0-15
Hasegawa T	2010	GKRS	22	NA
Hasegawa T	2015	SRS	27	NA
Iwai Y	2003	GKRS	11	NA
Iwai Y	2005	GKRS	14	NA
Jang N	2010	EBRT	70	1.8
Jiang GL	1994	EBRT	NA	NA
Kim JO	2013	FSRT	50.4	1.8
Kocher M	2013	FSRT	52.2	1.8
Kountouri M	2019	PBRT	74	1.8-2.0
Kumre K	2015	EBRT	NA	NA
QT Le	2003	EBRT +SRT	66	1.8/ 7-15
Leavitt JA	2013	GKRS	18	NA
Li PC	2019	Proton	70	1.46-2.0
Liao H	2014	FSRS	21	7
Mackley Hb	2007	IMRT	45.9	1.7-2.0
Madani I	2009	IMRT	70	2.0
Marchetti M	2016	MGKR	25	5.0
Midena E.	1987	EBRT	70	N/B
Midena E.	1987	EBRT	65	N/B
Miller RC	1997	SRS	15	2.0
Minniti G	2007	EBRT	NA	1.8
Morange- Ramos I	1998	GKRS	28	N/B
Nakamura	2016	PBRT	70.4	2.2
Nutting N	1999	EBRT	NA	1.8
Ove R	2000	GKRS	13	NB
Ozkaya A	2017	EBRT	NA	2.0-2.12
Paek SH	2005	FSRT	50	1.8
Park S	2017	EBRT	NA	2.0
Paulino A.C.	1989	EBRT	61.7	1.8-2.0
Pigeaud Klessens MLE	1992	EBRT	62	N/B
Pollock BE	2014	SFSR	32	N/B
Puataweepong	2015	FSRT	45	2.3
Puataweepong	2015	SRS	16.8	N/B
Rajan B	1993	EBRT	56	1.52
Repka MC	2018	EBRT +boost 10 Gy	60	2.0
Roa WH	1994	EBRT	68.4	1.8-2.0
Rosenblatt E	2003	EBRT	66.45	1.8-2.0
Skeie BS	100	GKRS	33.1	NA
Stafford SL	2003	EBRT	50.2	N/B
Takeda A	1999	EBRT	NA	1.2-2.0
Thompson	1983	EBRT	65.9	N/B
Van Balen	1988	EBRT	67.7	N/B
van den Bergh	2004	EBRT	NA	1.8-2.0
van den Bergh	2003	EBRT	49.5	1.8-2.1
Wang	2018	HFSR	18.2	NA
Weber DC	2006	SSPT	69.9	1.6-1.8
Kothe	2021	PBRT	70-74	1.8-2.0
Zijing LI	2021	PBRT	NA	N/B
Yan-Ling Wu	2021	IMRT	NA	N/B
Puyao C Li	2019	EBRT+PBRT	70	1.9
Sukwoo Hong	2021	SRS	25	N/B
Rabih Hage	2021	PBRT	52.4	1.8
Kuen Tze Lin	2019	EBRT	52.2	1.8
Thingthing Zhang	2021	IMRT	60.37	N/B
Thingthing Zhang	2021	IMRT	57.01	N/B
Hiroaki Sufuji	2018	CIR	NA	N/B
Gishan Ratnayak	2019	SRS	50.4	1.8
Toshihiko Inoue	2018	IMRT	52.8	N/B

.

Abbreviations used in the table:
EBRT= external beam radiation therapy
PBRT= proton beam radiation therapy
MGKR= multisession gamma knife radiosurgery
FSRT= fractionated stereotactic radiation therapy
IMRT= intensity modulated radiation therapy
GKRS= gamma knife radiosurgery
SRS= stereotactic radiosurgery
FSRS= fractionated stereotactic radiosurgery
SFSR= single- fraction stereotactic radiosurgery
HFSR= hypofractionated stereotactic radiosurgery
SSPT= spot scanning proton therapy
CIR= carbon ion radiation therapy
